# Items and Remarks

**Published:** 1873-09

**Authors:** E. N. Brush


### Items and Remarks.

BY E. N. BRUSH.

In some remarks, made in assuming the chair at the recent meeting of the Buffalo Medical Association, to consider the sanitary condition of the city, Prof. JAS. P. WHITE, so well expressed the sentiments of the majority of those present that we present our readers with an abstract thereof, as we are unable to give a full report of the meeting. He said:

"The medical man is and should be looked upon as a guardian of the public health. He should consider it his duty, to warn the people against the approach of any destructive epidemic, and to teach them by a proper observation of sanitary rules to ward off its fatal results. During the five epidemics of cholera through which I have passed in this city since 1832 and of which I am, I believe, the only surviving physician then in practice, I have observed, that until the authorities and citizen learned that only by putting the city in a correct sanitary condition could the inroads of cholera be prevented, that the mortality was frightful. When however they came to realize the fact that filth and squallor were its chief auxillaries, and took proper measures to insure cleanliness, and proper hygienic laws were enforced, a marked decrease was noticed both in the number of cases and in the mortality from cholera What have we then learned from these five epidemics? We have learned to prevent in a large degree the approach of cholera, by hygienic means, and when it does appear, by prompt and active measures to save the life of the patient. The subject of prompt attention to those attacked with cholera should be brought before the people, for it is only in the early stages of the attack that we can hope to be of benefit to the patients, and they should realize the importance of calling their physician as soon as the first symptoms present themselves.

In our desire to place our city in a good sanitary condition we must not overlook the fact that personal cleanliness is as much to be desired as the cleanliness of our surroundings, and this fact should be urged upon the citizens.

We have learned to prevent the approach of cholera by proper sanitary conditions and it now remains to be seen whether our city is in such condition.

I do not know that I have seen the streets in a more filthy condition for years than they were in the early spring and summer months. Much has been done to obviate this condition, yet much remains to be done. The contractors should be made to understand that the filth must be removed. I am pleased to observe that within the past few weeks the Board of Health have been making active exertions to place the city in a better condition as regards cleanliness. They should be sustained and encouraged in their endeavors.

One, of the many nuisances of which I might speak, is the practice of wetting or sprinkling the streets when in a filthy condition. There could scarcely be suggested a practice which seems so harmless, and yet is so full of pernicious results. It is well known that as a rule dry dirt is innocuous but as soon as it is placed in a favorable condition, one of moisture, a process of fermentation takes place, disease germs are produced and the poisonous emenations are exhaled to contaminate the air. The wood pavements are in this manner rendered an active source of miasm The broken wood fiber, and excrementitious animal matter, when damp and exposed to heat, rapidly undergoes decomposition, and imperceptibly poisons the atmosphere. The habit of sprinkling the yards and sidewalks at night is especially bad, as the rays of the sun are then lacking to cause rapid evaporation and the surroundings remain damp and unhealthy during the night, creating an unhealthy and miasmatic atmosphere. Another source of contamination, which I am glad to see is to be abated, and to which I can but refer, is the swill nuisance. In many localities the swill and garbage is thrown into the streets and alleys, presenting an active source of disease. It is to be hoped that the conditions of the present contract will be strictly enforced. The privy vaults in certain portions of our city are placed upon the surface, and a person can on nearly all occasions recognize the effluvia arising therefrom. This is a matter of which the Board of Health should take cognizance, and all owners of badly sewered or overflowing vaults should be made at once to abate the nuisance.

The present indications are that cholera in its onward march may visit Buffalo, unless we prepare ourselves against it. It may be guarded against by proper hygienic measures, and the authorities should be urged to immediate and prompt action. They should be encouraged and sustained in their efforts thus far, and the citizens informed of the necessity of co-operating with them in the work of putting the city in a proper sanitary condition.

Should the association think it proper, a committee might be appointed to advise and co-operate with the Board of Health in their efforts.

The negligence of the proper persons has left many of the nuisances unabated, which now at this season it would be injudicious to attempt to remove. Much however can yet be done. The experience of other cities has demonstrated that by hygienic means cholera can be policed and kept at bay, and it remains for Buffalo to add another to the list of examples, proving that epidemic cholera can be prevented.

—At the fire in Boston, of May 30, *Dr. Jourdain's Gallery of Anatomy*, was destroyed. This institution was a typacle one of its kind. At a meeting of the Boston Society for Medical Observation, resolutions were passed petitioning his Honor the Mayor to refuse to grant a new lisence to the proprietor. In his reply to the committee appointed to communicate with him, the Mayor said that the proprietor of the *Gallery* had not applied for a new lisence, but that if he did, such application would be met with a prompt refusal whenever it was made. He also informed the Society, that he had refused a license recently for a similar institution on Court street. This is really as the *Boston Medical and Surgical Journal* styles it "A Triumph of Morality."

—The managers of the Harrisburg, Pa., Hospital are in trouble, the attending staff of the institution having in a body resigned. The cause of the trouble arose from the attempt of the Board to introduce Homœopathy into the institution. Two physicians on the Board of Managers also resigned. The physicians were sustained in their action by the County Medical Society, the members individually pledging themselves not to accept any position in the Hospital until the old staff was reinstated. It looks as if the managers would have to adopt Homœopathy entirely or loose the one hundred dollars which they invested in a case of Homœpathic remedies.

—*The Philadelphia Medical Times* in speaking of the case of Absence of the Uterus, recently reported in this Journal, and the bad treatment which it received previous to falling into Prof. White's hands says: "Our cotemporary who publishes this case, discreetly, and perhaps wisely, gives only the initials of the first two medical attendants; but it seems a shame that such monstrous and inexcusable ignorance can not be exposed, so that it may at least be avoided." We claim for our profession that during the past century we have made rapid and wonderful advancement in our means of diagnosis and methods of treatment, and pride ourselves upon the certainty with which we can detect the seat of anatomical lesions; yet were we to give the names of those whose "monstrous and inexcusable ignorance" we frequently observe, we should be in continual hot water. What would the Editor of the *Times* say on observing a case of Congenital Hernia tapped for Hydrocele, and a case of fractured femur with four inches shortening, treated for one week with liniments, etc, as a simple contusion. Yet both these cases fell under our observation recently in one day. Fortunately in the one case the child was placed upon its back when the operation was made and the Hernia reduced itself before the trocar was inserted. In the other case the patient did not live to ask any *disagreeable* questions.——The Annual Announcement of the College of Physicians and Surgeons of Syracuse, says: "The course of Opthalmology will consist of lectures on the Anatomy, Physiology and Diseases of the Ear. ——The report of the meeting of the American Institute of Homœophathy in the *Cincinnati Medical Advance*, affords some most amusing reading. Evidently the editor could not find much in the papers read before the Institute to comment on, so he turns his attention to the members themselves, and speaks of different members after the following manner, giving their names in each instance, the sagacious man, the radical man, the disputatious man, the earnest man, the silent man, the funny man, the serious man, the scientific man, the talkative man, the handsome man, the polite man, and so on. We notice in this report that the paper of Dr. Gregg, of this city, was voted stale. Some of the papers were spoken of as valuless, others as shooting over the heads of some of the members, and the whole meeting according to the *Advance*, was characterised by too much talk. The statement of Dr. Gilchrist, of Tidioute, Pa., that Homœopathy can cure every known disease, called forth an incredulous smile. It certainly seems a little boastful to say the least.——The same Journal speaks of the failure of an effort to throw overboard the election of one of the board of censors, because she happened to be a lady. We suppose if *she* had happened to have been a gentleman it would have been all right. ——Articles two and four in the original department of this number were read at the meeting of the Medical Association of Central New York, in June, 1872.——A new Journal, or rather an old one revised, the *Peninsular Journal of Medicine*, has made its appearance. We welcome it to our exchange list.

